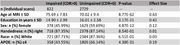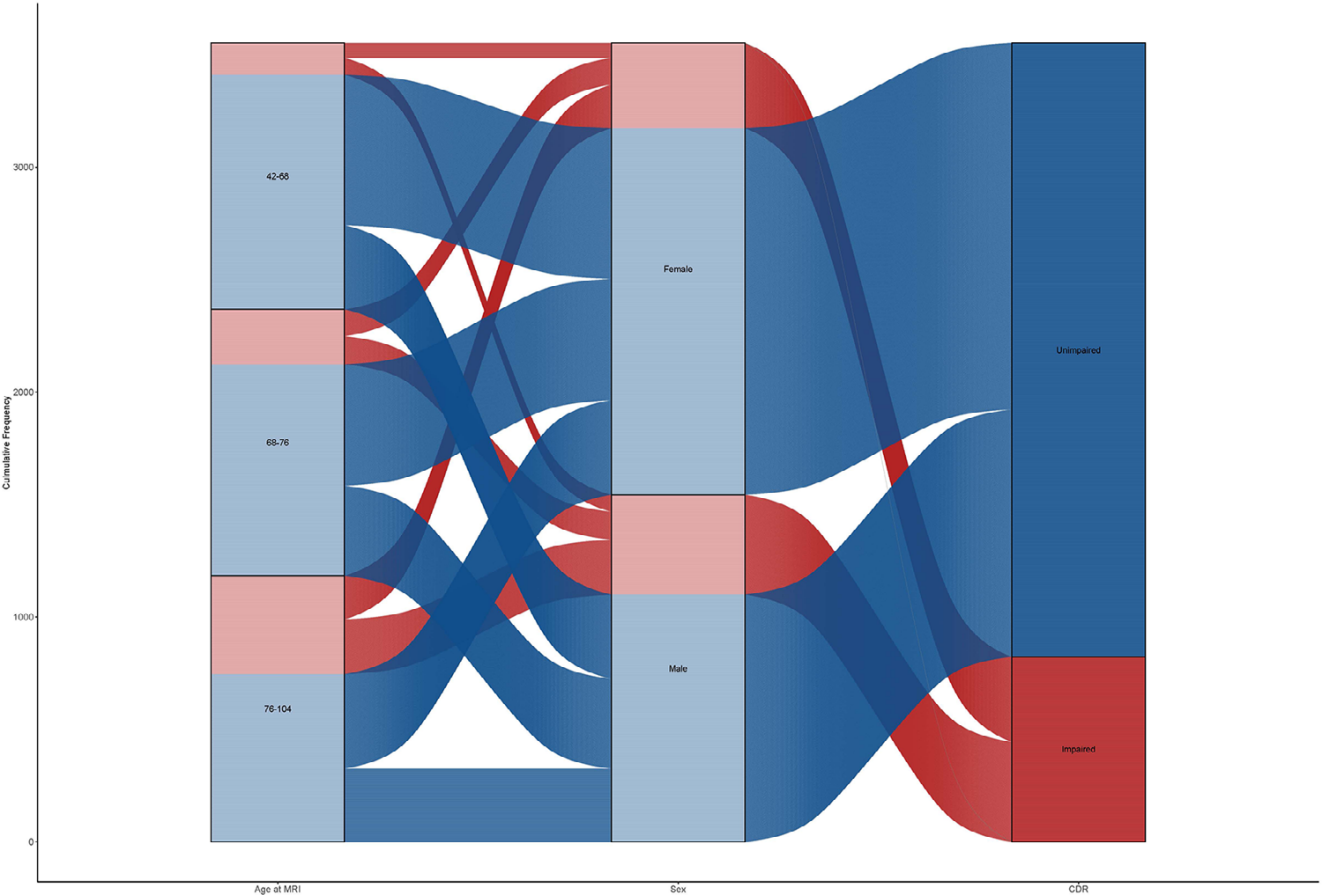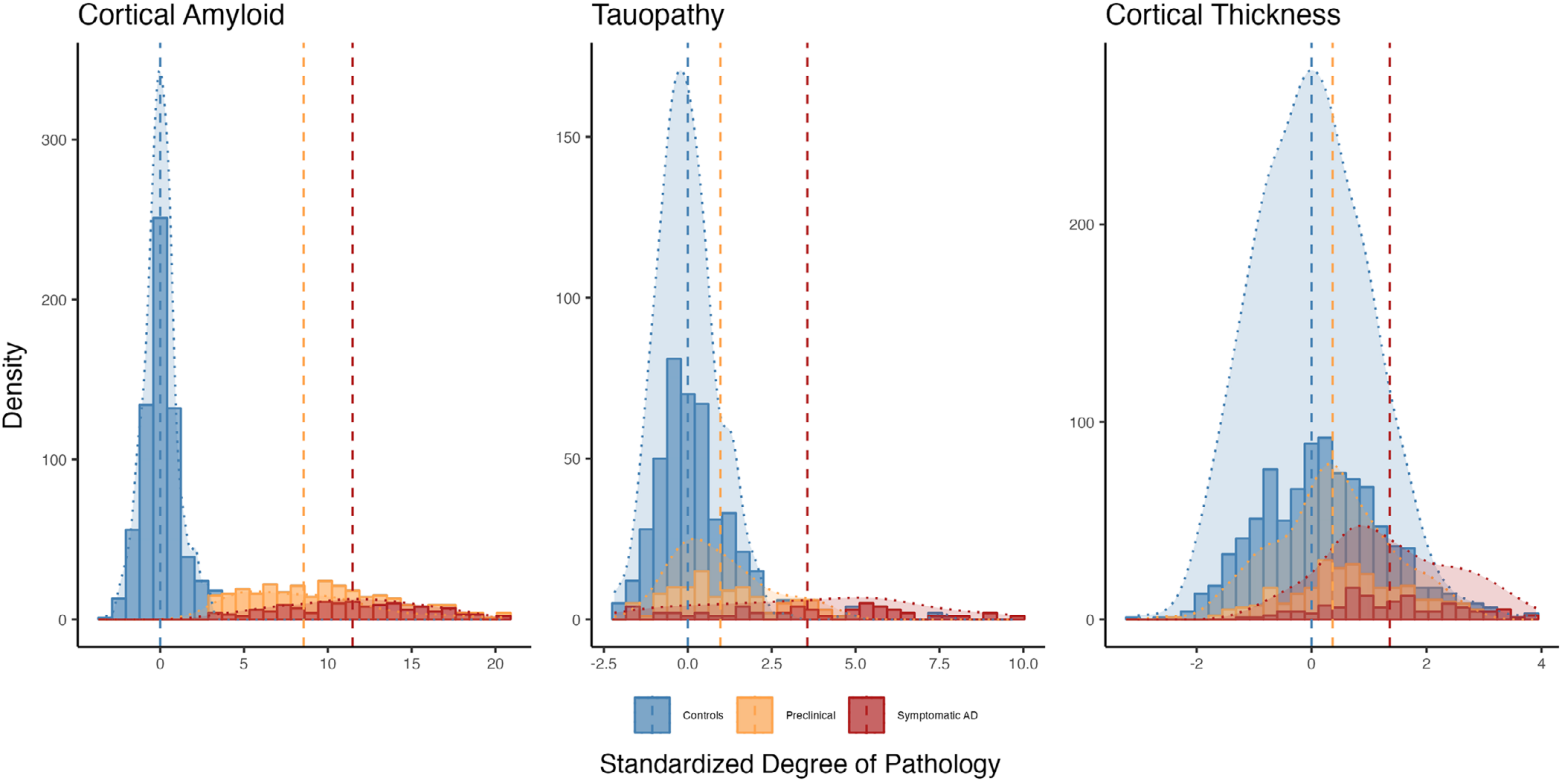# The Knight Alzheimer Research Imaging dataset: a comprehensive multimodal resource for exploring aging, preclinical, and symptomatic Alzheimer disease pathology

**DOI:** 10.1002/alz.086955

**Published:** 2025-01-09

**Authors:** David A. Hoagey, Nicole S. McKay, Brian A. Gordon, Austin A. McCullough, Suzanne E. Schindler, Chengjie Xiong, John C. Morris, Tammie L.S. Benzinger

**Affiliations:** ^1^ Washington University in St. Louis School of Medicine, St. Louis, MO USA; ^2^ Washington University in St. Louis, St. Louis, MO USA; ^3^ Washington University in St. Louis, School of Medicine, St. Louis, MO USA

## Abstract

**Background:**

The Knight Alzheimer Research Imaging (KARI) dataset, a compilation of data from projects conducted at Washington University in St. Louis, represents a comprehensive effort to advance our understanding of Alzheimer disease (AD) through multimodal data collection. The overarching goal is to characterize normal aging and disease progression to contribute insights into the biological changes preceding AD symptom onset.

**Methods:**

The dataset comprises cross‐sectional and longitudinal measures of magnetic resonance imaging (MRI), including T1 and T2‐weighted structural, diffusion, and resting‐state acquisitions, from over 1,600 participants aged 42 to 103 years. Additionally, a subset of participants underwent amyloid (1,100+) and tau (500+) tracer positron emission tomography (PET) acquisitions, along with cognitive, genetic, and cerebrospinal fluid (CSF) biofluid measures.

**Results:**

In addition to raw MRI and PET acquisitions, this dataset also includes processed output that has undergone rigorous quality assessment, anatomical segmentation, and quantification procedures using FreeSurfer, as well as post‐processing quantification of amyloid and tau burden. All data are publicly available in an online repository and can be accessed upon request.

**Conclusions:**

The KARI dataset offers a valuable resource for investigating biomarkers, genetic factors, and imaging data in normal aging, preclinical, and symptomatic Alzheimer’s disease. Expanded use could contribute to the development of early diagnostic tools, treatment strategies, and a deeper understanding of the complexities associated with neurodegenerative diseases.